# Acute Myeloid Leukemia in a Cholecystectomy Specimen

**DOI:** 10.1155/2022/2956052

**Published:** 2022-09-26

**Authors:** Ana I. Hernandez Caballero, Eduardo Castro-Echeverry, Roula Katerji, Christopher Gonzalez

**Affiliations:** ^1^Department of Pathology, Baylor Scott and White Healthcare, Temple, TX, USA; ^2^Department of Pathology and Laboratory Medicine, University of Rochester Medical Center, Rochester, NY, USA

## Abstract

A 76-year-old man was admitted into the ER for upper abdominal pain. Physical exam and CT scan confirmed acute cholecystitis with multiple cholelithis, and a cholecystectomy was performed. The cholecystectomy specimen showed chronic cholecystitis with exuberant inflammatory infiltrate. On careful examination of the specimen, large atypical cells with vesicular chromatin, folded nuclei, and inconspicuous red nucleoli were noted percolating into the gallbladder wall and lining vascular spaces. These cells were positive for CD117, CD43, and myeloperoxidase and negative for CD20 and CD3 stains. Further workup including peripheral blood flow cytometry confirmed a population of circulating immature myeloid precursors comprising about 38% of events. This is a rare case of acute myeloid leukemia that came to clinical attention by incidentally involving the gallbladder.

## 1. Introduction

Blood-based processes such as leukemia and lymphoma can be incidentally found in a myriad of pathology specimens. The best-known examples are those of chronic lymphocytic leukemia involving specimens such as colon resections [1]. Here, we present an uncommon case of acute myeloid leukemia incidentally found to be involving a cholecystectomy specimen.

## 2. Case Presentation

A 76-year-old man was admitted into the ER for severe upper abdominal pain. Physical exam and CT scan confirmed acute cholecystitis with multiple choleliths, and an emergent cholecystectomy was performed.

**Figure 1:**
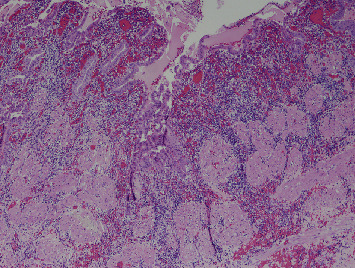
Original magnification ×40 hematoxylin and eosin stain, depicting thickened muscularis propria with an area of mucosa herniation through the muscularis propria (Rokitansky-Aschoff sinuse) and accompanying exuberant inflammatory infiltrate.

**Figure 2:**
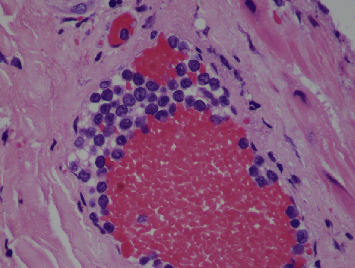
Original magnification ×400 hematoxylin and eosin stain, depicting a mural vessel with intraluminal large mononucleated cells with occasional folding of the nuclear membranes, open chromatin, and an inconspicuous red nucleoli, representing immature precursors.

**Figure 3:**
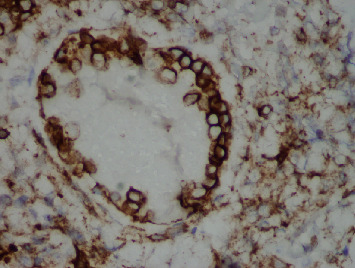
Original magnification ×400 CD43 immunohistochemical stain, depicting CD43 membranous staining of the intravascular immature precursors.

**Figure 4:**
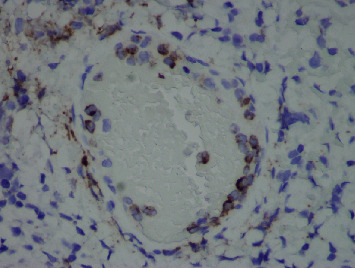
Original magnification ×400 myeloperoxidase immunohistochemical stain, depicting characteristic granular cytoplasmic staining of myeloperoxidase in the intravascular immature precursors.

Gross exam of the specimen showed multiple granular gallstones measuring up to 1.5 cm and a thickened gallbladder wall that measured up to 1 cm. On microscopic exam, the specimen showed chronic cholecystitis with exuberant inflammatory infiltrate (Figure 1). On careful examination, atypical cells with vesicular chromatin, folded nuclei, and inconspicuous red nucleoli were noted percolating into the gallbladder wall and lining vascular spaces (Figure 2). These cells were positive for CD117, CD43 (Figure 3), and myeloperoxidase (Figure 4) stains and negative for CD20 and CD3 stains. These findings were concerning for a possible acute leukemia or myeloid sarcoma. After communicating with the clinical team, a peripheral blood smear was reviewed and flow cytometry studies were performed on peripheral blood.

Peripheral blood smear review confirmed the presence of numerous circulating blasts showing high nuclear to cytoplasmic ratio; fine, powdery chromatin with one or more prominent nucleoli; and scant blue cytoplasm. No Auer rods were seen. Flow cytometry verified the presence of a myeloid blast population representing 38% of events, with coexpression of CD13, CD33 (dim), CD117, HLA-DR and myeloperoxidase and negative for CD34, CD3, CD7, CD11b, CD14, CD16, CD19, CD56, CD10, and TdT. Additionally, review of the patient's electronic chart showed that at presentation to the ER the patient's WBC was 79.5 × 10*e*9/L, hemoglobin 7.1 g/dL, and platelet count 37 × 10*e*/L.

Upon further questioning of the patient and his family, it was found that the patient did have a history of acute myeloid leukemia, diagnosed overseas. Review of the outside medical records indicated that the patient had a bone marrow biopsy showing 86% blasts. Karyotype could not be performed and molecular studies, at his institution, detected a FLT3 mutation. He had received one week of chemotherapy and G-CSF, four months prior to his presentation at our institution.

## 3. Discussion

This is a very interesting and easy-to-miss case of acute myeloid leukemia presenting in a cholecystectomy specimen. The key finding that prompted further assessment of the specimen was the large, atypical intravascular cells. A limited panel of stains that included CD20, CD3, CD43, CD117, and myeloperoxidase was performed aimed at classifying these cells. The main differential diagnosis for this large, atypical intravascular infiltrate included (1) intravascular large B-cell lymphoma, (2) immature lymphoid/myeloid neoplasms such as acute myeloid leukemia and acute lymphoblastic leukemia, and (3) myeloid sarcoma.

Intravascular large B-cell lymphomas represent a rare extranodal type of large B-cell lymphoma characterized by selective growth of neoplastic cells within the lumina of vessels [2, 3]. It usually affects patients in their sixth to seventh decade of life. However, intravascular large B-cell lymphoma clinical presentation and behavior differ according to patients' geographical origin [4]. In Western populations, it usually presents with symptoms related to the main organ involved, in contrast to Asian populations, where it presents with a multiorgan failure and pancytopenia picture. Histologically, the neoplastic cells are mainly lodged in the lumen of small- and intermediate-sized vessels. Cytologically, the tumor cells are large with prominent nucleoli and frequent mitotic figures.

Intravascular large B-cell lymphoma cells express mature B-cell antigens (such as CD19, CD20, PAX5, and CD22). CD5 and CD10 coexpression is seen in 38% and 13% of the cases, respectively [3].

Acute lymphoblastic leukemia/lymphoma includes neoplasms of B- and T-cell differentiation. Generally, acute lymphoblastic leukemia/lymphoma is a disease of children and adolescents, although it can present in adulthood as well. In adulthood, it is more likely to be of T-cell differentiation. Acute lymphoblastic leukemia/lymphoma have a relatively low propensity of leukemic presentation being more commonly found involving nodal and extranodal sites [5, 6] such as the central nervous system, spleen, liver, and testes. Cytologically, the neoplastic cells present scant cytoplasm with dispersed nuclear chromatin with multiple nucleoli. Immunophenotypically, acute lymphoblastic leukemia/lymphoma cells usually express markers of immaturity, such as TdT and CD99 [7], and lineage differentiation markers such as CD3, CD2, CD4, CD5, CD7, and CD8 (T-cell markers) and CD10, PAX5 [8], and CD20 (variable) (B-cell markers).

Myeloid sarcoma is defined as a tumor mass comprised of myeloid blasts occurring outside of the bone marrow. Tissue infiltration by myeloid blasts in a patient with leukemia is not classified as myeloid sarcoma unless it presents with tumor masses in which the tissue architecture is effaced [9]. In this case, although the myeloblasts infiltrate the gallbladder wall, they do not efface the gallbladder architecture. Rare cases of myeloid sarcoma involving the gallbladder in nonleukemic patients have been described [10, 11]. A recommended panel to approach cases suspicious of myeloid sarcoma is CD43, lysozyme, myeloperoxidase, CD68 (or CD163), CD117, CD3, and CD20 [12].

Acute myeloid leukemia was the leading differential diagnosis on this case. This entity presents at any age, but most patients are either infants or older adults. These neoplastic cells express markers of immaturity such as CD34 and CD117, as well as myeloid differentiation markers such as myeloperoxidase. Acute myeloid leukemias have an increasingly complex classification that includes molecularly defined entities. In this case, the patient's intravascular and interstitial atypical infiltrate was positive on immunohistochemistry for CD117, CD43 and myeloperoxidase and negative for CD20 and CD3.

On this case, the lack of cytogenetic information precludes final classification of the acute myeloid leukemia. However, FLT3 mutations, regardless of the cytogenetics [13, 14], confer a shorter disease-free survival [13, 15] and, in many instances, a worse overall survival [14, 15].

In summary, this case highlights some of the morphologic clues of blasts on hematoxylin and eosin stain (high nuclear to cytoplasmic ratio, vesicular chromatin, folded/irregular nuclei, and inconspicuous red nucleoli), reviews some of the key markers and considerations for the differential diagnosis of intravascular hematolymphoid cells, and presents an uncommon example of a hematolymphoid neoplasm inadvertently involving a specimen retrieved for unrelated reasons illustrating the intricacy of a second diagnosis in pathology.

## Data Availability

The data described can be found in our department's archives.
